# The impact of cardiopulmonary exercise testing (CPET) and Charlson comorbidity index (CCI) in a large contemporary cohort of patients undergoing robot‐assisted radical cystectomy and intracorporeal urinary diversion (RARC‐ICUD)

**DOI:** 10.1002/bco2.191

**Published:** 2022-10-07

**Authors:** Arthur McPhee, Alexander Ridgway, Thomas Bird, Raj Pal, Edward W. Rowe, Anthony J. Koupparis, Jonathan J. Aning

**Affiliations:** ^1^ Bristol Urological Institute, North Bristol NHS Trust Southmead Hospital Bristol UK; ^2^ Department of Urology Addenbrooke's Hospital Cambridge UK; ^3^ Bristol Haematology and Oncology Centre University Hospitals Bristol NHS Foundation Trust Bristol UK; ^4^ Population Health Sciences, Bristol Medical School University of Bristol Bristol UK

**Keywords:** bladder cancer, Charlson comorbidity index, complication, risk prediction, robot‐assisted radical cystectomy

## Abstract

**Objective:**

The aim of this study was to investigate whether pre‐operative comorbidity status measured by the Charlson comorbidity index (CCI) or cardiopulmonary exercise testing (CPET) is associated with postoperative complications and length of stay (LOS) in patients undergoing robot‐assisted radical cystectomy and intracorporeal urinary diversion (RARC‐ICUD).

**Patients and methods:**

We conducted a retrospective study of a prospectively maintained database of 428 consecutive patients who underwent RARC‐ICUD at a tertiary referral centre between 2011 and 2019. CCI was correlated with peri‐operative outcomes including postoperative LOS, Clavien–Dindo (CD) complications and survival. A planned subgroup analysis was performed to evaluate the relationship between pre‐operative CPET, and the same outcomes utilising the threshold of anaerobic threshold (AT) ≥ 11/ <11 ml/kg/min were analysed.

**Results:**

Of the total cohort, 350 patients undergoing RARC‐ICUD with complete data were included in the final analysis. A CCI score ≥5 was associated with a higher rate of CD III–V complications at 30‐day incidence rate ratio (IRR) = 3.033, (*p* = 0.02) and at 90‐day IRR 2.495, (*p* = 0.04) postsurgery. LOS was not associated with CCI; the strongest association with LOS was a CD complication of any grading. CCI did not predict readmission or mortality rates after surgery. Subanalyses of patients who underwent pre‐operative CPET found that CPET <11 ml/kg/min did not predict for LOS, CD complications or death within 1 year of surgery.

**Conclusions:**

CCI score is a simple, reliable and cost‐effective way of identifying patients at increased risk of complication after RARC‐ICUD. Surgeons performing radical cystectomy should consider utilising CCI to augment pre‐operative patient counselling prior to RARC‐ICUD.

## 1 INTRODUCTION

The gold standard treatment for muscle‐invasive bladder cancer (MIBC) remains radical cystectomy with bilateral pelvic lymph node dissection and urinary diversion.^1^ Open radical cystectomy has been associated with an overall complication rate of 47%–68% and a Clavien–Dindo (CD) ≥3 complication rate of 9%–21% at 90 days postoperatively.^2^, ^3^, ^4^, ^5^ Although minimally invasive techniques for performing radical cystectomy have recently been adopted with the aim of reducing patient morbidity and enhancing recovery, randomised controlled trials and meta analyses of data to date demonstrate similar complication rates to the open approach.^6^, ^7^, ^8^

Robot‐assisted radical cystectomy and intracorporeal urinary diversion (RARC‐ICUD) is acknowledged to be technically challenging to perform but the most minimally invasive surgical approach. RARC‐ICUD and the increasing adoption of robotic surgery have led to older, more comorbid patients with bladder cancer being considered for surgery.^9^ Recent data comparing intracorporeal and extracorporeal urinary diversion (ECUD) outcomes showed that highly comorbid patients undergoing ICUD had a lower risk of postoperative complications than those undergoing ECUD.^10^ Nevertheless, complications occurred with both techniques.

There is a need for better tools to facilitate patient counselling about individualised risks of surgery and to enable healthcare professionals to more accurately predict outcomes after RARC‐ICUD, especially in more comorbid populations. Although risk prediction tools have shown utility in patients undergoing open radical cystectomy,^11^, ^12^ their ability to predict outcomes in patients undergoing RARC‐ICUD is currently poor. One of the most widely accepted objective discriminatory measures for assessing patients' risk of increased length of stay (LOS) and rate of major complications is cardiopulmonary exercise testing (CPET).^13^ However, CPET has been shown to be less discriminatory in predicting outcomes when studied in patients undergoing RARC‐ICUD.^14^

The Charlson comorbidity index (CCI) is an established validated method of predicting long‐term survival in an unselected population. The CCI assigns a weighted score to 19 comorbidities; the total score corresponds to a percentage 10‐year mortality prediction. CCI has been assessed as a predictive tool for postoperative outcomes in gastrointestinal (GI), gynaecological and orthopaedic surgeries^15^, ^16^, ^17^ but has not been extensively studied in patients undergoing RARC‐ICUD.

The purpose of this study was to evaluate whether pre‐operative comorbidity status measured by the CCI was associated with LOS, complications and survival at up to 1 year after surgery in a large series of patients undergoing RARC‐ICUD at a tertiary referral centre with an established enhanced recovery programme (ERP). In addition, we aimed to compare the performance of CCI with CPET in our cohort.

## 2 PATIENTS AND METHODS

**FIGURE 1:**
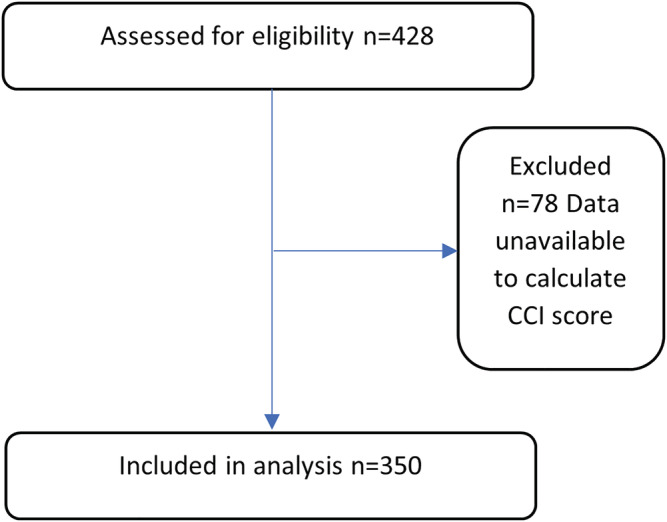
Study CONSORT flowchart

A consecutive series of 428 patients undergoing RARC‐ICUD for bladder cancer from our institution's prospectively maintained electronic cystectomy database was analysed. These patients underwent RARC‐ICUD between April 2011 and October 2019. All RARC‐ICUD surgeries were performed by four surgeons DG, AK, EWR and JA (AK, EWR and JA were fellowship trained surgeons) with high volume experience in robotic surgery (each performing at least 100 robot‐assisted radical prostatectomy procedures per year) prior to the first RARC‐ICUD procedure recorded. No procedure was converted to open; each surgeon performed more than 15 RARC‐ICUD per year over the duration of the study and submitted their outcome data to the BAUS Oncology database. Our database did not include CCI scoring at its inception, so this was retrospectively calculated from patients' pre‐operative medical records using the weighted index of comorbidities.^18^ Where incomplete information meant a score could not be calculated, patients were excluded from analysis. A total of 78 patients were excluded from final analysis. Figure 1 shows a flow diagram of patients included in the study.

During the study period, all patients undergoing RARC‐ICUD at our institution underwent pre‐operative assessment by a consultant anaesthetist. CPET was requested routinely for patients listed for RARC‐ICUD; however, CPET was only mandated as part of the pre‐operative assessment process if the anaesthetist or surgeon felt this was necessary. Patients undergoing RARC‐ICUD in this study did not undergo a prehabilitation programme, and CCI score did not contribute to the decision to perform CPET testing. The CPET result did not directly influence the peri‐operative management plan of patients undergoing RARC‐ICUD. All patients undergoing RARC‐ICUD were managed according to a standardised peri‐operative enhanced recovery protocol with no additional pathway incorporated based upon the results of the CPET test.

Demographic variables including age, sex, height and weight were recorded; in addition, pre‐operative characteristics such as age, American Society of Anaesthesiologists (ASA) score, diabetes, smoking history, pre‐operative haemoglobin (Hb) and eGFR, previous abdominal surgery, previous radiotherapy, CPET and the use of neo‐adjuvant chemotherapy were reviewed.

### 2.1 Statistical analysis

Previous studies have demonstrated that 10‐year survival decreases rapidly once a patient's CCI score increases and patients with a CCI score ≥5 are considered high risk.^18^, ^19^, ^20^ This metric has been used in other studies assessing cystectomy.^20^ For this reason, we performed an analysis using the cutoff of ≥5 to create groups for comparison. This was done after using SPSS to perform ROC curves confirmed a CCI of 5 was an appropriate cutoff for our dataset.

We investigated the significance of pre‐operative CCI score on LOS, CD complication rates at 30 and 90 days and mortality rates at 30 days, 90 days and 1 year after surgery. Additionally, we correlated demographic and pre‐operative factors including age, ASA, gender, BMI, neo‐adjuvant chemotherapy and pre‐operative anaemia to LOS using Spearman's correlation. Further analysis was then performed using independent *t*‐tests and negative binomial regression analysis (NBRA), which has been used to study the link between CCI and LOS previously.^21^ All comparative tests were two‐tailed unless otherwise stated, and statistical significance was set at *p* = 0.05. A planned subgroup analysis was performed to evaluate the relationship between pre‐operative CPET and postoperative outcomes. Patients were divided into two cohorts for the CPET risk stratification evaluation. Those with an anaerobic threshold (AT) of <11 ml/kg/min and those with an AT ≥ 11 ml/kg/min.^22^ All statistical analyses were performed using SPSS version 25 (IBM, USA).

## 3 RESULTS

### 3.1 Demographic

In total, 350 patients, mean age 67.5 years (range 18–89), with complete CCI data underwent RARC‐ICUD during the study period. Of these, 259 (74%) were male, and the majority, 260 (62%), were ASA 2 although 126 (36%) were ASA 3 or 4. Table 1 summarises the demographic of the included cohort. The mean CCI score for the cohort was 4.73 (range 2–9).

**TABLE 1:** Baseline demographic of study population (*n* = 350)

Demographics	
Age, mean (range)	67.5 (41–89) years
Male (%)	259 (74%)
Female (%)	91 (26%)
BMI, mean (range)	27 (17–44)
ASA score, *n* (%)	
1	7 (2)
2	217 (62)
3	125 (35.7)
4	1 (0.28)
Charlson comorbidity index (CCI), *n* (%)	
0–3	60 (17.1)
4–6	260 (74.2)
7+	30 (8.6)
Mean	4.73
Neoadjuvant chemotherapy, *n* (%)	
Complete course	85 (24.3)
Incomplete course	16 (4.6)
No	249 (71.1)
Clinical T‐stage, *n*	
1	116
2	183
3	36
4	15
Pathological N‐stage, *n*	
0	234
1	23
2	32
3	3
X	58
Surgical time, min	
Mean	235
Pelvic lymph node dissection, *n*	
None	30
Standard	320
Urinary diversion type, *n*	
Ileal conduit	332
Orthotopic substitute	18
Length of stay	
Median	7 days
Mean	9.15 days
Range	3–77 days

### 3.2 Peri‐operative outcomes

The mean LOS for the cohort was 9.15 days (range 3–77), and 31 (9%) of patients were re‐admitted to the hospital within 90 days of discharge. In total, 162/350 (46%) patients suffered CD I–V complications and 35/350 (10%) CD III–V complications within 30 days of surgery. When CD complication rates within 90 days of surgery were evaluated, 169 (48%) and 40 (11%) sustained CD I–II complications and CD III–V complications, respectively. About 4/350 (1%) patients died within 30 days of surgery and 10/350 (3%) within 90 days of surgery. At 1 year, 95/350 (27%) had died after their surgery.

### 3.3 Analysis of cohort outcomes by CCI score

Table 2 illustrates the cohort LOS and complications data split by CCI score <5 and ≥5. Patients undergoing RARC‐ICUD with a CCI score of ≥5 had a higher LOS than those with a CCI score of <5 (*p* = 0.047, one‐tailed *t*‐test).

**TABLE 2:** Length of stay and complication rates among CCI score groups

CCI score	No. of patients, *n*	Mean LOS, days	Median LOS, days	Mean no. of 30 days complications CD I–V	Mean no. of 90 days complications CD I–V	Mean no of 30 days complications CD III–V	Mean no. of 90 days complications CD III–V
<5	154	7.98	6	0.5	0.53	0.10	0.11
≥5	196	9.43	7	0.9	0.97	0.24	0.29

Abbreviations: CD, Clavien–Dindo; CCI, Charlson comorbidity index; LOS, length of stay.

When examining the rate of CD complications, patients with CCI scores ≥5 had a higher number of CD I–V and CD III–V complications at 30 and 90 days than those with a CCI score of <5 (Table 2). Independent *t*‐tests demonstrated a significant difference for CD I–V and CD III–V complication rates at both 30 days (*p* = 0.004, *p* = 0.039) and 90 days (*p* = 0.004, *p* = 0.025) between these two groups.

Using NBRA for complication data, we found that CCI ≥ 5 was associated with an increase in CD I–V complications at 30 days and an increase in CD III–V complications at both 30 and 90 days. This was statistically significant in all cases. For CD III–V complications, the IRR for patients with a CCI score ≥5 was 3.053, (*p* = 0.01) at 30 days and 2.51 (*p* = 0.026) at 90 days, demonstrating an increased risk of major complications in the order of ×3 at 30 days and ×2.5 at 90 days, (Tables 3a and 3b). There was no association between complication rates and any other peri‐operative factor, except intraoperative blood loss which had an insignificant effect size of 1.002 (*p* = 0.001).

**TABLE 3a:** Negative binomial regression analysis of CD III–V complications for RARC patients at 30 days

Peri‐operative characteristic	IRR	95% confidence interval	*P* value
CCI ≥ 5	3.053	1.299–7.174	0.01
ASA score	0.819	0.477–1.408	0.822
Pre‐operative anaemia	1.494	0.784–2.847	0.233
Neo‐adjuvant chemotherapy	1.211	0.632–2.320	0.564
Age	0.966	0.957–1.035	0.822
Final pathological stage	0.886	0.680–1.154	0.368
BMI	0.942	0.875–1.106	0.120
Duration of surgery	0.999	0.994–1.004	0.698
Prior pelvic radiotherapy	0.834	0.170–4.085	0.822
Intraoperative blood loss	1.002	1.000–1.003	0.01

Abbreviations: ASA, American Society of Anaesthesiologists; CD, Clavien–Dindo; CCI, Charlson comorbidity index; IRR, incidence rate ratio; RARC, robot‐assisted radical cystectomy.

**TABLE 3b:** Negative binomial regression analysis of CD III–V complications for RARC patients at 90 days

Peri‐operative characteristic	IRR	95% confidence interval	*P* value
CCI ≥ 5	3.053	1.299–7.174	0.01
ASA score	0.819	0.477–1.408	0.822
Pre‐operative anaemia	1.494	0.784–2.847	0.233
Neo‐adjuvant chemotherapy	1.211	0.632–2.320	0.564
Age	0.966	0.957–1.035	0.822
Final pathological stage	0.886	0.680–1.154	0.368
BMI	0.942	0.875–1.106	0.120
Duration of surgery	0.999	0.994–1.004	0.698
Prior pelvic radiotherapy	0.834	0.170–4.085	0.822
Intraoperative blood loss	1.002	1.000–1.003	0.01

Abbreviations: ASA, American Society of Anaesthesiologists; CD, Clavien–Dindo; CCI, Charlson comorbidity index; IRR, incidence rate ratio; RARC, robot‐assisted radical cystectomy.

There were no statistically significant associations between CCI score and readmission rates or mortality.

NBRA demonstrated that several factors can increase the risk of a prolonged hospital stay (Table 4). Unsurprisingly, 30‐day CD complications of any grade significantly increased risk of a longer hospital admission, incidence rate ratio (IRR) 1.597 (*p* = <0.001) for CD 1–2 complications and IRR 2.181 (*p* = <0.001) for CD 3–5 complications; prior pelvic radiotherapy and pre‐operative anaemia also marginally increased the risk of an increased LOS, IRR 1.35 (*p* = 0.03) and IRR 1.121 (*p* = 0.012), respectively. Age, duration of surgery and intraoperative blood loss were all significant but had insignificant effect sizes, while ASA score, neo‐adjuvant chemotherapy, T‐stage and BMI bore no association.

**TABLE 4:** Negative binomial regression analysis of LOS for RARC patients

Peri‐operative characteristic	IRR	95% confidence interval	*P* value
CCI	0.951	0.901–1.003	0.066
CCI ≥ 5	1.061	0.930–1.211	0.378
ASA score	1.014	1.004–1.017	0.733
Pre‐operative anaemia	1.121	1.026–1.226	0.012
Neo‐adjuvant chemotherapy	0.999	0.907–1.100	0.980
Age	1.011	1.004–1.017	0.001
Final pathological stage	0.987	0.951–1.025	0.507
BMI	1.002	0.992–1.011	0.681
Duration of surgery	1.001	1.001–1.002	<0.001
Prior pelvic radiotherapy	1.325	1.100–1.595	0.03
Intraoperative blood loss	1.000	1.000–1.001	0.01
30‐day Clavien–Dindo I–II complication	1.597	1.465–1.741	<0.001
30‐day Clavien–Dindo III–V complication	2.181	1.958–2.430	<0.001

Abbreviations: ASA, American Society of Anaesthesiologists; CCI, Charlson comorbidity index; IRR, incidence rate ratio; LOS, length of stay; RARC, robot‐assisted radical cystectomy.

### 3.4 Subgroup analysis of cohort outcomes by CPET

Of the cohort in whom CCI was calculated, 188 had a CPET performed. In the planned subgroup analysis of CPET association with outcomes, an additional 32 patients in whom we could not calculate the CCI were included. These 220 patients were categorised into groups using the AT value <11 or ≥11. Table 5 summarises the cohort LOS and complications data.

**TABLE 5:** CPET LOS and complication metrics

Anaerobic threshold (AT)	No. of patients, *n*	Mean LOS, days	Median LOS, days	Mean no of 30 days complications CD I–V	Mean no. of 90 days complications CD I–V	Mean no. of 30 days complications CD III–V	Mean no. of 90 days complications CD III–V
<11	66	8.06	7	0.77	0.85	0.15	0.20
≥11	154	8.34	7	0.58	0.63	0.11	0.14

Abbreviations: CD, Clavien–Dindo; CPET, cardiopulmonary exercise testing; LOS, length of stay.

No significant difference in LOS, CD complication rates at 30 and 90 days from surgery or 1 year survival was identified when cohorts were stratified by AT >/<11. We explored whether using lower AT values to stratify patients undergoing RARC‐ICUD would alter these findings but did not identify any signal that lower values would be associated with poorer outcomes. No correlation between CPET AT measurements and the CCI scores in this cohort was identified. NBRA did not show any correlation between CPET AT and LOS or CD complications at 30 and 90 days.

## 4 DISCUSSION

Minimally invasive techniques have offered the potential to reduce the physiological impact of radical cystectomy for patients with bladder cancer. However, such approaches are still associated with morbidity, and patients with bladder cancer are often a comorbid group. Current methods of risk prediction for minimally invasive surgery are recognised to be in need of improvement.^23^, ^24^, ^25^, ^26^, ^27^ This study has demonstrated that a CCI threshold of ≥5 is associated with a higher rate of postoperative complications at 30 and 90 days after RARC‐ICUD. CPET did not predict for complications in our RARC‐ICUD population. Furthermore, our analysis demonstrates that the most strongly associated factor with increased LOS after RARC‐ICUD is the presence of a complication.

Accurate risk prediction is essential to guide patient counselling and facilitate shared decision‐making. Recent analysis of UK data suggests that a significant proportion of patients with localised MIBC may not be receiving radical treatment,^28^ and it may be that inaccurate risk prediction is playing a role in patients not being offered definitive treatment. Early modelling attempts used postoperative characteristics to risk stratify patients undergoing open cystectomy, but this was recognised as having limited utility in counselling pre‐operative patients.^27^ Successful risk prediction models used in other specialities have failed to translate successfully to cystectomy. Golan et al. studied the ACS‐NSQIP risk calculator in cystectomy patients. The ACS‐NSQIP calculator was created after modelling 2.4 million general surgical cases over 4 years; however, when applied retrospectively to 954 cystectomy patients, it poorly predicted risk of postoperative complications.^23^ This highlighted the challenges of nonprocedure specific risk prediction models. The POSSOM system (Pre‐operative score to predict postoperative mortality) described recently by Froehner et al.^11^, ^12^ has demonstrated some promise in patients undergoing open radical cystectomy but in the most recent analysis ASA class was shown to be the most powerful single predictor of 90‐day mortality in patients undergoing open radical cystectomy. In the present study of RARC‐ICUD, we found that ASA did not have predictive ability for postoperative complications. Previous studies examining CCI utility in risk prediction after radical cystectomy have included cohorts with patients that have undergone both open and minimally invasive surgeries^29^ or ICUD and ECUD.^10^ CCI has also previously been used to ensure that radical cystectomy cohorts studied are adequately matched.^30^ Our study is unique in that it examines a large contemporary cohort of exclusively RARC‐ICUD patients and focuses on correlating CCI with short‐term and medium‐term outcomes of interest to clinicians and patients.

Contrary to our previous work and the work of others in smaller predominantly open radical cystectomy populations,^13^, ^31^ we did not find that CPET predicted for outcomes in our larger exclusively RARC‐ICUD population. Although our findings may be limited by our subgroup analysis approach, the limitations of CPET in predicting outcomes in robotic cystectomy have been identified in other cohorts.^18^ We hypothesise that CPET may not be predictive of outcomes in RARC‐ICUD due to reduced morbidity associated with the intracorporeal approach. The iROC study recently reported that RARC‐ICUD resulted in a statistically significant increase in days alive and out of hospital over 90 days after surgery in addition to improved early qualitative recovery compared with open radical cystectomy.^32^ While the iROC data illustrate that RARC‐ICUD is likely to be associated with lower morbidity, the study did not include CPET in the analysis; this is an evidence gap that needs to be filled. CPET is an expensive resource; however, it does provide objective physiological data which can be used to monitor or measure response to prehabiliation interventions.^33^ This may be an area where CPET provides added value in the care of patients identified to be at high risk prior to RARC‐ICUD.

Pragmatically, risk prediction modelling also facilitates patient preparation before surgery and ensures appropriate postoperative support resources are available. Moving forwards our institution will incorporate CCI to identify patients at higher risk of complications and as a consequence a potentially longer postoperative stay. This will have the benefit of allowing us to plan more effectively with regard to critical care utilisation, bed occupancy and the cost of delivering care. CCI has been explored by other surgical specialties and has been found to be useful in predicting LOS and adverse events. CCI has been investigated for morbidity and mortality in GI cancer, ovarian cancer and oesophageal cancer,^15^, ^16^ with its role being explored in noncancer surgery such as orthopaedics.^17^, ^18^, ^19^, ^20^, ^21^ Each of these studies supports the use of CCI to predict the risk of adverse events. Given that CCI has demonstrated its utility across different fields, its ease of use and reliance on wholly pre‐operative data, we believe that it should be incorporated into the workup and assessment of RARC patients.

Our present study has several limitations: We acknowledge that this is a single centre retrospective series and our findings need to be validated in other centres. A weakness is that CCI scores were calculated retrospectively from pre‐operative patient medical records and therefore could be subject to misclassification bias; however, we excluded all patients who underwent RARC‐ICUD in whom the CCI could not be explicitly calculated from the final analysis to minimise this risk. Due to our large cohort, we would not expect the distribution of CCI scores in the patients excluded from the final analysis to be different. Additionally, this study includes a large cohort including cases performed during and after the learning curve, and therefore, we believe that the findings are representative of real‐life practice. In our cohort consistent with UK practice, the majority of patients underwent an ileal conduit urinary diversion.^34^ While we do not believe that our findings would be different in centres with a higher proportion of patients undergoing neobladder formation, this is a potential area for future study. Our CPET findings are hypothesis generating but merit further investigation in a blinded randomised study as they could be subject to selection bias, as we accept that not all patients in our cohort underwent a CPET; some bladder cancer patients with poor CPET results potentially may not have proceeded to undergo surgery and thus will not have been captured in this study. While CCI is an easy to measure pre‐operative score that demonstrates utility, it was introduced in 1987 and was not specifically intended for the evaluation of patients undergoing RARC‐ICUD. Further work is needed to improve risk prediction in this population. In future work, we will aim to develop a specific predictive model by increasing the size of our datasets and including additional variables such as performance status and frailty.

## 5 CONCLUSION

The Charlson comorbidity score is a simple, reliable and cost‐effective approach to identifying patients at increased risk of postoperative complications following RARC‐ICUD. CPET should not be used as a ‘rule out’ investigation in patients otherwise considered eligible for RARC‐ICUD. Surgeons performing robot‐assisted radical cystectomy should consider utilising CCI as a tool to tailor pre‐operative patient counselling, improve shared decision‐making and service planning.

## ETHICS STATEMENT

Ethics approval was not applied for or required for this study. The study was performed in accordance with the Declaration of Helsinki.

## DISCLOSURE OF INTEREST

The authors report no relevant financial conflict of interests.

## AUTHOR CONTRIBUTIONS

Study concept and design: J Aning and A McPhee. Acquisition of data: A Ridgway, A McPhee, and T Bird. Statistical Analysis: A McPhee. Analysis and interpretation of the data: A McPhee, J Aning, and A Koupparis. Drafting of the manuscript and critical revision: McPhee, Ridgway, Bird, Pal, Rowe, Koupparis, and Aning.
